# Dietary Intake Patterns, Substance Use and Their Association with Anxiety and Depression Symptoms in Medical Students in Mexico: A Cross-Sectional Study

**DOI:** 10.3390/nu18010104

**Published:** 2025-12-28

**Authors:** Linet Arvilla-Salas, Sodel Vazquez-Reyes, Alfredo Salazar de Santiago, Leticia A. Ramirez-Hernandez, Idalia Garza-Veloz, Fabiana Esther Mollinedo-Montaño, Celia Luna-Pacheco, Francisco Luna-Pacheco, Margarita L. Martinez-Fierro

**Affiliations:** 1 Doctorado en Ciencias con Orientación en Medicina Molecular, Unidad Academica de Medicina Humana y Ciencias de la Salud, Universidad Autonoma de Zacatecas, Carretera Zacatecas-Guadalajara Km 6, Ejido la Escondida, Zacatecas 98160, Mexico; nutrimentdmm@gmail.com (L.A.-S.); idaliagv@uaz.edu.mx (I.G.-V.); fabiana.mollinedo@uaz.edu.mx (F.E.M.-M.); 2 Unidad Académica de Odontología, Universidad Autonoma de Zacatecas, Zacatecas 98160, Mexico; asalazar@uaz.edu.mx (A.S.d.S.); celialuna@uaz.edu.mx (C.L.-P.); pacolunap@uaz.edu.mx (F.L.-P.); 3 Unidad Academica de Matematicas, Autonomous University of Zacatecas, Paseo la Bufa, Av. Solidaridad, Zacatecas 98066, Mexico; lramirez@uaz.edu.mx

**Keywords:** ultra-processed foods, unprocessed or minimally processed foods, food frequency questionnaire, anxiety, depression, consumption patterns, medical students

## Abstract

**Background/Objectives**: The growing prevalence of mental health problems among medical students is a global concern, with dietary patterns emerging as potential modifiable factors. This study aimed to explore and evaluate whether higher consumption of ultra-processed foods may be associated with greater symptoms of anxiety and depression. **Methods**: This was an exploratory cross-sectional study integrated into a previous cohort of medical students, conducted based on the guidelines for Strengthening the Reporting of Observational Studies in Epidemiology. Sixty-seven medical students completed a Food Frequency Questionnaire-based index. Dietary patterns and the associations between these patterns and symptoms of anxiety and/or depression were assessed statistically. **Results**: There were differences in the consumption of unprocessed or minimally processed foods, including fruits, vegetables, legumes and unsweetened juices between groups with/without anxiety or depression (*p* < 0.05). A higher intake of ultra-processed foods such as pizza, hot dogs, cereals high in fat and sugar, processed beverages and sweets was linked to greater symptoms (*p* < 0.05; Cohen’s *d* = 0.3–0.7). Three to four dietary patterns were identified, explaining between 60% and 86% of the variance. High consumption of cereals with added fat and sugars increased the risk by 7.4 times (OR = 7.4, 95% CI 1.2–12.2, *p* < 0.05). **Conclusions**: Dietary intake was associated, but not causally linked, to emotional symptoms among medical students. Lower consumption of unprocessed foods and higher intake of ultra-processed foods formed consistent behavioral profiles associated with anxiety and depression. Consuming more than three daily servings of cereals with added fat and sugar increased the risk of severe depressive symptoms by more than sevenfold, highlighting a strong dietary determinant. Future research should assess nutritional interventions aimed to improve mental health and academic performance in medical students.

## 1. Introduction

Mental disorders are a major challenge in global public health, with increasing rates across various population groups [1]. In this context, the mental health of medical students is a key concern due to the demanding nature of their academic and professional training. The intense curriculum, ongoing competition, long hours of study and clinical work and continuous exposure to emotionally stressful situations create an environment that greatly impacts the psychological well-being of these future healthcare professionals [2]. Multiple studies indicate that this group faces a high prevalence of psychiatric symptoms, such as stress, anxiety, depression, bipolar disorder, attention deficit/hyperactivity disorder (ADHD) and others [2,3,4]. According to the Pan American Health Organization (PAHO), one in four people in the Americas experiences mental illness at some point in their lives [5]. 

The etiology of mental disorders is multifactorial and involves a complex interaction between genetic and environmental components, where genetic heritability explains only about 40% of cases [6,7]. Environmental factors such as chronic stress, early adverse experiences and, more recently, diet have been identified as relevant modulators at the brain level. From a neurobiological perspective, it has been proposed that nutritional imbalances due to poor nutrition and a lack of essential nutrients [8,9,10,11] can induce systemic inflammatory responses, including the release of proinflammatory cytokines such as tumor necrosis factor alpha (TNF-α), interferon gamma (IFN-γ), interleukin (IL) 1 and IL-6, negatively affecting brain function and the regulation of mood, appetite and sleep [6,12,13].

Nutrition has become increasingly important in understanding, preventing and treating mental disorders because eating habits can affect mental health [6,7,14,15,16,17,18,19]. The International Society for Nutritional Psychiatry Research has emphasized the need to incorporate nutritional medicine into psychiatric practice, based on evidence linking dietary patterns with neurobehavioral changes [20,21]. Diets high in ultra-processed foods, saturated fats and added sugars, along with a low intake of fruits, vegetables, legumes and essential micronutrients, have been associated with metabolic problems and a higher incidence of symptoms of depression and anxiety or other mental disorders [20,22,23,24,25]. These dietary habits are linked to lower levels of neurotransmitters such as serotonin and dopamine, among others [12,26,27,28]. Several international studies support this association; in the United States (US), lower fruit/vegetable consumption and higher added sugar intake were linked to increased anxiety and depression in college students (especially males) [29]. In Iran, a plant-based diet was inversely associated with depressive symptoms [30]. In India, students without symptoms had higher-quality diets during the pandemic [31], whereas in England and Turkey, greater nutritional knowledge and adherence to the Mediterranean diet were correlated with lower levels of anxiety and depression [32].

A study on Iranian medical students found a direct link between unhealthy dietary patterns and a higher risk of depression [33]. In Mexico, although some studies have investigated the presence of depressive and anxiety symptoms in university students and their relationship with specific eating behaviors [34], the overall dietary patterns associated with anxiety and depressive symptoms in this population have not been comprehensively examined. Therefore, the present observational study aims to explore and evaluate whether a higher consumption of ultra-processed food (UPF) could be associated with greater symptoms of anxiety and depression.

## 2. Materials and Methods

### 2.1. Study Design and Participants

**Figure 1:**
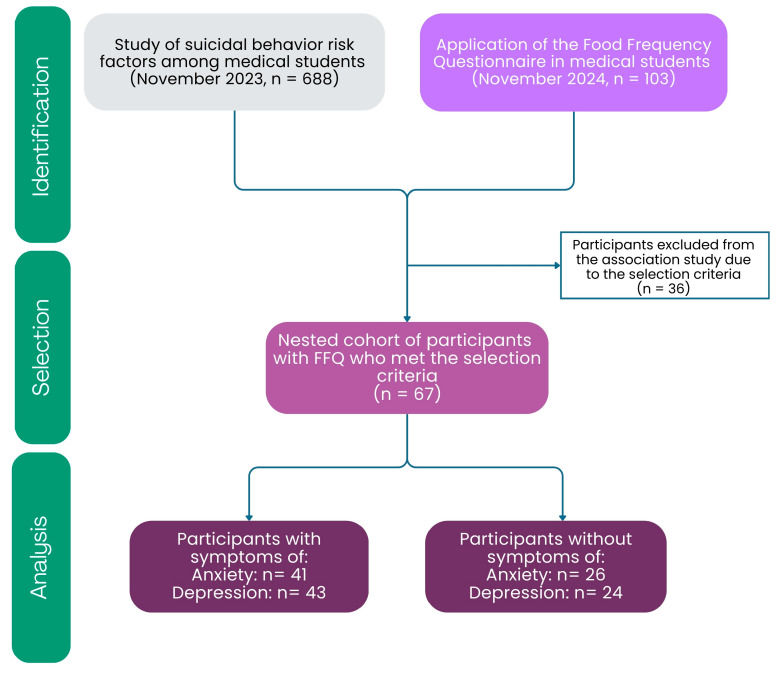
Flowchart of the study population. The study began with an initial cohort of 688 students from the Academic Unit of Human Medicine and Health Sciences. Participants in this cohort already had mental health data collected in 2023 [4]. In November 2024, an aleatory cohort of students from the same Academic Unit completed the FFQ from the ENSANUT. Subsequently, both cohorts were aligned for the association study between FFQ and anxiety and/or depression symptoms (this study), in which 67 participants met the selection criteria. During the analysis stage, these participants were classified as having or not having anxiety and/or depression symptoms. A total of 36 students were excluded because they had no data on mental health. The figure breaks down the total sample (n = 67) independently for each condition. For anxiety, 41 participants with symptoms and 26 without symptoms were grouped together; for depression, 43 participants showed symptoms, and 24 were without symptoms. Thus, the sum of each category reflects the total number of participants evaluated according to the presence or absence of symptoms.

A previous study on risk factors for suicidal behavior was conducted among medical students at the Academic Unit of Human Medicine and Health Sciences of the Autonomous University of Zacatecas. It was a cross-sectional study nested within a previous cohort of medical students. In that cohort of students (n=688), mental health variables were measured in 2023 [4]. Subsequently, in November 2024 (n=103), a Food Frequency Questionnaire (FFQ) of the National Health and Nutrition Survey (ENSANUT) was validated and applied (Figure 1) [34]. The FFQ was given in the classrooms of the study participants with support from the responsible teachers.

For the association study, those students who had both mental health data and FFQ data were selected (n=67). The students selected for this part of the study met the following criteria: enrollment in the Academic Unit of Human Medicine and Health Sciences; age between 18 and 26 years; and previous participation in a study on risk factors for suicidal behavior (Figure 1).

All participants provided informed consent for their participation and did not receive any compensation for taking part in the study. No additional exclusion criteria were established, as all students who voluntarily participated and fully completed the required instruments were included in the analysis.

### 2.2. Instruments and Data Collection Procedure

For the study of risk factors associated with suicidal behavior, various instruments were used as part of the primary research, including the Beck Suicidal Ideation Scale, the Depression, Anxiety, and Stress Scale (DASS-21) [35], the International Physical Activity Questionnaire (IPAQ) [36], the Alcohol Use Disorders Identification Test (AUDIT) [37], the Cannabis Addiction Test (CAST) [38], the Drug Use Situations Inventory (DUSI) [39] and the Reasons for Attempting Suicide Questionnaire (RASQ) [40], among others. Detailed information on reliability, validity, scales and the different inventories used in this study can be found in our previous study, Martinez-Fierro et al. (2025) [41]. This reference provides the necessary methodological foundation to support the reliability of the measurement tools used. The FFQ used was based on ENSANUT [34].

#### 2.2.1. Instrument Categorization

Suicidal behavior was categorized into three groups: “normal,” “assessment” and “treatment,” according to the classification proposed in the Beck Suicidal Ideation Scale [42]. These categories reflect clinical decision thresholds indicating the absence of suicidal ideation (“normal”), the need for further clinical evaluation (“assessment”) or the need for immediate clinical intervention (“treatment”), rather than levels of symptom severity. In contrast, anxiety and depression were classified as “normal,” “mild,” “moderate” and “severe” according to the DASS-21 [35]. Physical activity levels were categorized as “low,” “medium” and “high” based on the IPAQ [36]. Consumption situations according to the DUSI were categorized “N/A” (not applicable, no consumption), “<50%” (consumption in less than 50% of assessed risk situations) and “>50%” (consumption in more than 50% of such situations) [39]. Alcohol use was categorized as “low risk,” “medium risk,” “high risk” and “probable addiction” according to the AUDIT classification [37]. Cannabis use was classified as “without symptoms of addiction,” “with symptoms of problematic use” and “symptoms of addiction” based on the CAST [38]. 

FFQ was analyzed based on the daily intake of unprocessed or minimally processed foods such as fruits, vegetables and legumes, as well as ultra-processed foods—including snack-type dairy products (sweetened yogurt, flavored milk, among others), fast food, cereals with added fats and sugars, snacks, sweets and desserts, along with nutritional supplements. The frequency of consumption was converted to equivalent daily servings; to do this, sections of the FFQ (days/week, times/day and number of servings) were used to estimate the average daily intake of each food. The classification followed the NOVA food classification system [43], which categorizes foods according to their level of processing and nutritional content. The FFQ was organized into nine food groups and reported consumption quantities: “dairy”; “fruits”; “vegetables”; “fast food”; “legumes”; “cereals”; “beverages”; “snacks, sweets, and desserts”; “supplements”; and “reported consumption amount.” The FFQ’s response options, drawn from ENSANUT [34], included days of the week: “0”, “1”, “2”, “3”, “4”, “5”, “6” and “7”. Additionally, a section indicated how many times the food was consumed per day: “0”, “1”, “2”, “3”, “4”, “5” and “6.” There was also a section with portion sizes: “0”, “1”, “2”, “3”, “4”, “5” and “6.” It is important to note that because the purpose of this exploratory study was to characterize the range of dietary behaviors associated with emotional symptoms in medical students, ultra-processed food (UPF) exposure was analyzed in a disaggregated manner rather than through a single continuous NOVA-based score. This approach allowed us to preserve the diagnostic specificity of both protective (NOVA I) and high-risk (NOVA IV) food groups, which would not be adequately captured by an aggregated UPF index.

#### 2.2.2. Food Frequency Questionnaire

The FFQ was generated using a Google Form (https://shre.ink/qgW3 (accessed on 3 November 2025)) and validated in two phases: the first involved content validity, and the second included criterion validity and reliability. For content validity, a panel of ten experts was selected based on their professional experience, specialty and academic degree. The criteria assessed were clarity of writing, clarity of instructions, internal consistency, appropriate language, whether the instrument measures what it aims to measure and how well it fulfills its stated purpose. These experts also evaluated the logical order of the questions and the adequacy of the items to gather the desired information, as well as provided additional comments. A QR code linking to the FFQ was sent via WhatsApp to the group of experts for online evaluation, including the instrument’s evaluation criteria. Aiken’s V validity coefficient and Lawshe’s content validity ratio were calculated. To assess criterion validity and reliability, data from a sample of thirty students who completed the instrument were analyzed. It was validated using Cronbach’s α and the intraclass correlation coefficient.

### 2.3. Ethical Considerations

The protocol was reviewed and approved by the Institutional Ethics and Research Committees on 12 May 2023, with registration numbers AMMCCI-FACTOR-06 and CEICANCL-12052023. Students received detailed information about the study and read the informed consent form, and those who agreed to participate signed their informed consent in the initial section of the questionnaire. International ethical standards, institutional guidelines and compliance with the General Health Law regarding research were followed, classifying it as low-risk research. The International Ethical Guidelines for Health Research Involving Human Subjects, the Declaration of Helsinki and the STROBE guidelines were also followed [44]. Furthermore, since the studies were conducted using online surveys, they were designed to comply with the Federal Law on the Protection of Personal Data Held by Private Parties to prevent indirect harm to participants and safeguard their data.

### 2.4. Statistical Analysis

Descriptive statistics were used to summarize the demographic, clinical and dietary data. Continuous variables were expressed as means ± standard deviations (SDs), while categorical variables were expressed as frequencies and percentages. Between-group comparisons (e.g., presence versus absence of anxiety or depression) were performed using chi-squared tests for categorical variables and Student’s *t*-tests or Mann–Whitney U-tests for continuous variables, depending on whether the distributions were normal. To assess group differences across dietary items, a multivariate analysis of variance (MANOVA) was performed. Where statistically significant differences were found, effect sizes were calculated using Cohen’s d. Pearson’s correlation coefficients were used to explore associations between continuous variables such as the frequency of food group intake and DASS-21 scores. To reduce dimensionality and identify underlying dietary patterns, principal component analysis (PCA) with varimax rotation was conducted within symptom severity subgroups. The adequacy of the data was assessed using the Kaiser–Meyer–Olkin (KMO) test and Bartlett’s test of sphericity. In addition, ordinal logistic regression was performed to identify potential associated factors of symptom severity (four levels for depression and a binary outcome for anxiety). Variables that had been considered significant in previous analyses were included as associated factors (e.g., consumption of specific food groups), and cannabis use and comorbid depression were added as covariates where appropriate. Demographic variables, such as sex and age, were not included as covariates in the multivariate models, as no significant differences were found between groups in the bivariate comparisons. The study employed a convenience sampling strategy, recruiting medical students who voluntarily agreed to participate; therefore, a priori sample size estimation was not applicable. To enhance transparency regarding the study’s statistical sensitivity, post hoc power analyses were conducted using simulations based on the observed frequency of depression (22%) and anxiety (30%), as well as the distribution of key dietary predictors (e.g., 33% reported consuming cake or pie on two or more days per week). All analyses were performed using IBM SPSS Statistics (v.29), and *p*-values < 0.05 were considered statistically significant.

## 3. Results

### 3.1. Validation of the Food Frequency Questionnaire

The FFQ validation included two stages (content validity and criterion validity and reliability). For the content validity, a panel of ten experts evaluated the FFQ instrument (see Methods section for additional details). The results of this stage showed an Aiken’s V validity coefficient of 0.93 (v=0.93; 95% CI:0.8–0.97) and Lawshe’s Content Validity Ratio of 0.88 (CVR = 0.88). To assess criterion validity and reliability, data from a random sample of thirty medical students who completed the FFQ were considered. The results of this stage are displayed in Tables S1–S4 of the Supplementary Material. The Cronbach’s alpha value was 0.915 (α=0.915), and the intraclass correlation coefficient was 0.915 (r=0.915; 95% CI:0.866–0.953).

### 3.2. Description Charactetistics of the Study Population

Sixty-seven medical students were included in the study. To analyze the relationship between food consumption and mental health, DASS-21 scores were subgrouped into “presence” (clinical symptoms) and “absence” (normal). This subcategorization was performed to mitigate the complexity arising from the large number of variables and categories, allowing for the statistical modeling, reducing convergence errors and facilitating the clear identification of risk groups in the various analyses applied (Table 1).

The average age was 20.87 ± 1.585 years, and 59.7% were women (Table 1). A total of 47.8% n=32 of the participants were from outside the city, while 52.2% n=35 were originally from the area. Most students, about 83.6%, reported not working outside of school hours, whereas only 16.4% were employed. Of the students, 47.7% had high physical activity levels according to the IPAQ classification, followed by low (26.9%) and moderate levels (25.4%). Forty-one students (61.2%) exhibited symptoms of anxiety, and forty-three (64.2%) showed symptoms of depression. When comparing groups based on the presence or absence of anxiety, and separately on depression, no significant differences were found with respect to age, sex, being a foreigner, employment status or physical activity (*p* > 0.05). 

Most students, 80.6%, reported a low risk of alcohol use, followed by 17.9% with moderate risk and 1.5% with high risk (Table 1). Similarly, about 91% of students classified themselves as having “no symptoms of addiction” regarding cannabis use. These patterns may intersect with students’ eating behaviors, which are also shaped by emotional and social influences. Moreover, eating behavior may also be influenced by emotional factors; many people eat in response to emotional states (such as feeling sad or stressed; social pressure or happiness can also trigger eating). Alcohol and cannabis use often occur in social or emotional settings and tend to be linked with snack and ultra-processed food intake, creating a “recreational” pattern. Accordingly, we included situations of substance use and consumption to observe whether the intake of ultra-processed foods was mediated or accompanied by emotional states and/or substance use, which is important for exploring its relationship with anxiety and depression. When asked about the scenarios for substance use, the main reason was physical discomfort (38.8%), followed by pleasant moments (9%). Among the different situations, a significant association was found between depression and substance use during pleasant moments (*p* = 0.011). The results of substance use situations and their relationship to the presence or absence of symptoms of anxiety and depression are presented in Table S5 of the Supplementary Materials.

### 3.3. Food Consumption Frequency and Their Relationship with Anxiety and Depression Symptoms

The frequency of food consumption based on the total scores was examined in relation to the presence or absence of anxiety and depression symptoms. An interesting pattern appeared in the students’ dietary intake, characterized by two main observations: first, a clear specificity in the types of foods consumed; second, a difference in the consumption of unprocessed or minimally processed foods compared to ultra-processed products (Table 2). When comparing groups of students with and without anxiety symptoms, higher consumption of certain unprocessed or minimally processed foods was found among those without anxiety (*p* < 0.05). These foods included apples or pears, guava, red tomatoes, leafy greens, carrots, cucumbers, onions and lentils. In terms of ultra-processed foods, students with anxiety showed significantly higher consumption than non-anxious students of these products: pizza, hot dogs, fast food (at a daily frequency), doughnuts/churros, sugary cereals (e.g., sweetened flakes), cake/pie, caramelized popcorn, sweetened drinkable yogurt, soft drinks, sweetened natural juices and industrialized beverages with sugar (all p<0.05). 

On the other hand, when comparing students with and without depression, the only difference found was in the consumption of green leafy vegetables, which was higher in non-depressed students (p=0.012). It should be noted that within the group with anxiety, the foods with the highest consumption were boxed cornflake cereal (p=0.020), cookies of all kinds (p=0.050) and industrialized sugary drinks (p=0.030). The food group portions obtained per day from the FFQ are presented in the Supplementary Materials, Table S6. Regarding the different food groups, only the daily amount of beverage servings showed a significant difference between students with and without anxiety (*p* = 0.016; more servings in the group with anxiety). The rest of the food groups did not show statistically significant differences at the aggregate level (*p* > 0.05).

To facilitate the clinical interpretation of the findings obtained through MANOVA for each contrast, effect sizes (Cohen’s d) were calculated for the dietary differences observed between groups (Table 3). There were differences in weekly food consumption between students with and without anxiety symptoms. Within unprocessed or minimally processed foods, large negative effect sizes were observed, indicating markedly lower consumption among students with anxiety. This pattern was observed for apples or pears (*d* = −1.257; 95% CI: −2.35–−0.17), red tomato (*d* = −1.071; 95% CI: −2.20–−0.06), leafy greens such as chard, spinach or quelites (*d* = −1.560; 95% CI: −2.67–−0.45), carrots (*d* = −1.312; 95% CI: −2.52–−0.11) and onions (*d* = −1.095; 95% CI: −2.34–0.15). Moderate effects in the same direction were observed for guava (*d* = −0.832) and cucumber (*d* = −0.926). In contrast, lentils showed a positive effect size (*d* = 0.657), indicating higher intake among students with anxiety, while natural juice without added sugar showed only a small effect (*d* = 0.428). Ultra-processed foods demonstrated the opposite pattern, with most items showing positive effect sizes, consistent with higher consumption among students reporting anxiety. Notable examples included pizza (*d* = 0.660), bakery doughnuts and churros (*d* = 0.441), drinkable yogurt (*d* = 0.442), regular soda (*d* = 0.625; 95% CI: 0.002–1.25) and industrially processed sweetened beverages (*d* = 0.659). Smaller positive effects were observed for hot dogs, cake or pie, caramel popcorn, sweet cookies and both types of boxed cereal. Supplement use also showed a moderate positive effect (*d* = 0.707), suggesting greater use among students with anxiety. For depression, the pattern was similar but more selective. Students with depressive symptoms showed substantially lower consumption of leafy greens, with a large negative effect size (*d* = −1.521). Higher consumption of ultra-processed foods was also observed in this group, including cornflake cereal (*d* = 0.756), sweet cookies (*d* = 0.494) and industrially sweetened beverages (*d* = 0.464). Supplement intake showed a moderate positive effect as well (*d* = 0.625).

### 3.4. Correlation Analyses: Food Consumption, Substance Use and Menthal Health

**Figure 2:**
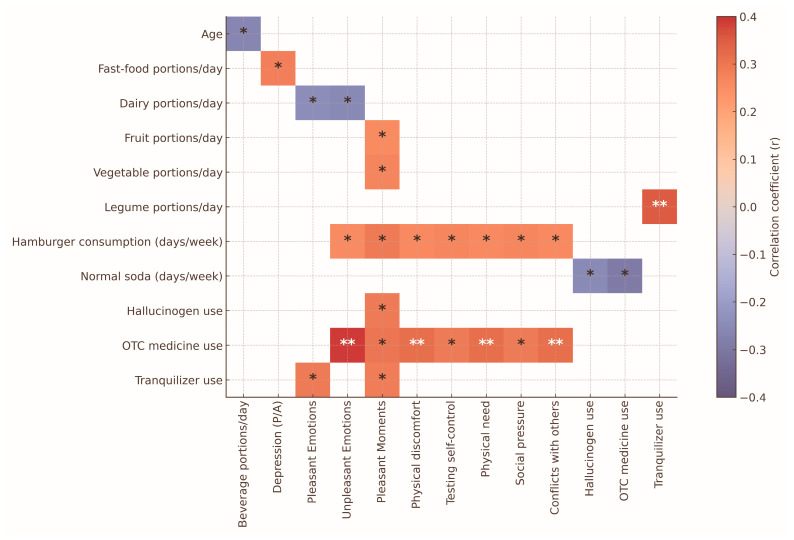
Heatmap of significant correlations among eating behaviors, substance use, substance use situations and psychological measures. The heatmap summarizes the correlations observed between dietary behaviors, substance-use patterns and emotional or psychological variables. Colors represent both the direction and magnitude of the associations: red tones indicate positive correlations, whereas blue tones reflect negative correlations; in both cases, color intensity corresponds to the strength of the correlation coefficient. Only statistically significant associations are marked within the cells, where one asterisk (*) denotes *p* < 0.05 and two asterisks (**) denote *p* < 0.01. Cells without asterisks indicate that no statistically significant correlation was detected. Abbreviations include P/A for “presence/absence” and OTC for “over-the-counter medication use”.

Pearson’s correlation analyses were conducted to explore associations between age, mental health scores (anxiety and depression), substance use and patterns of food consumption in specific emotional or social contexts. The results are presented in Figure 2. Age was negatively correlated with the frequency of sugary beverage intake (r=−0.263, p =0.032), suggesting that younger students reported higher consumption. Consumption of fast food was positively associated with depression scores (r=0.279, p =0.022). Additionally, the intake of ultra-processed foods such as hamburgers and sodas was positively correlated with emotional triggers—specifically “physical discomfort” (p < 0.05) and “social pressure” (p<0.05), as measured by items derived from the RASQ. In contrast, dairy product consumption showed no significant correlation with either pleasant or unpleasant emotional states, indicating a neutral pattern of emotional association (all r values close to zero). These findings suggest that specific ultra-processed foods may be consumed more frequently in contexts of negative affect or social influence.

### 3.5. Multivariate Analysis: Food Frequency and Symptoms of Anxiety and Depression

#### 3.5.1. Individual Foods

Based on the food identified as relevant in the preceding MANOVA test, logistic regression models were conducted to examine whether the number of times each food was consumed per week predicted the presence of anxiety or depression. In the models assessing anxiety, none of the foods reached conventional statistical significance; however, several showed noteworthy trends. Weekly onion consumption showed a marginal positive association with anxiety (β = 0.322, *p* = 0.087); therefore, it is observed that a higher frequency of consumption could be related to a higher probability of anxiety symptoms (see Table S7 of the Supplementary Materials).

Likewise, industrialized sugar-sweetened flavored beverages exhibited a near-significant positive association (β = 0.394, *p* = 0.073; Table S7). All other foods included in the anxiety models, such as apples/pears, guava, tomatoes, green leafy vegetables, baked goods, pizza, hot dogs, sweetened cereals and natural or industrialized juices, showed no significant associations. In contrast, within the models for depression, green leafy vegetables (chard, spinach, quelites) demonstrated a protective effect (β = −0.283, *p* = 0.030), suggesting that more frequent weekly consumption was associated with a lower likelihood of depressive symptoms. No additional foods showed significant associations in the depression models (Table 4).

#### 3.5.2. Food Group Consumption: Daily Portions and Daily Frequency

Additional logistic regression models were conducted to evaluate whether the number of daily portions and the number of times per day each food group was consumed predicted the presence of anxiety or depression. In the models based on daily portions, none of the food groups showed statistically significant associations with anxiety or depression. Nevertheless, several trends were observed. For anxiety, vegetable intake showed a nonsignificant inverse trend (β = −0.235, *p* = 0.141), whereas sweetened beverages approached marginal significance in a positive direction (β = 0.246, *p* = 0.108). For depression, the only food group showing a borderline trend was snacks, sweets and desserts, with higher daily portions indicating a potentially increased likelihood of depressive symptoms (β = 0.371, *p* = 0.088). No other food groups demonstrated meaningful associations at the portion level (Table S8 of the Supplementary Materials).

When examining the number of times per day each food group was consumed, results showed that legumes were the only significant predictor of anxiety (β = 0.402, *p* = 0.002), indicating that more frequent daily consumption was associated with a higher probability of reporting anxiety symptoms (Table 4). The remaining food groups, including fruits, vegetables, legumes, cereals, beverages and snacks, did not show significant associations with anxiety or depression. Across both modeling approaches, most food groups did not independently predict emotional symptomatology, though the consistent trends for sweetened beverages, snacks/desserts and fast food suggest potential dietary patterns that are worth exploring in larger samples (Table S9).

#### 3.5.3. Dietary Factors and Emotional Symptom Severity: Ordinal Regression Analysis

To further characterize the influence of dietary behaviors on the severity of emotional symptoms, ordinal logistic regression was performed, modeling depression and anxiety as ordered categories rather than binary outcomes. The model for depression demonstrated a statistically significant overall fit (*χ^2^*(17) = 42.9, *p* < 0.001; Nagelkerke R^2^ = 0.47), indicating that daily intake patterns contributed meaningfully to differentiating between severity levels (Table 5). In this model, consuming more than three daily portions of high-fat, high-sugar cereal products was strongly associated with increased odds of transitioning to a higher category of depressive symptoms (OR = 7.40, 95% CI: 1.19–12.15).

### 3.6. Identification of Dietary Patterns Related to Anxiety and Depression Using Exploratory Factor Analysis

Given that the bivariate analysis revealed several associations with anxiety and depression, but in the individual logistic regressions, it revealed mostly non-significant but suggestive trends (and many dietary variables showed overlapping directions), these items were described as probably reflecting broader dietary patterns rather than independent predictors. Additionally, substantial intercorrelations were observed among several consumption variables, indicating redundancy in how these foods contribute to overall eating behavior. To better explore this shared variance and observe significant composite patterns of intake, an exploratory principal component analysis (PCA) was performed.

In this stage, the FFQ data and the presence of anxiety and depression categorized by symptom severity (mild, moderate and severe) were used. The results are shown in Table S13 of the Supplementary Materials. The PCA revealed variable sampling adequacy across severity levels of anxiety and depression. For anxiety, KMO values ranged from low to moderate (0.326–0.580), with significant Bartlett’s tests in the mild (*χ*^2^ = 65.696, *p* = 0.0018) and moderate groups (*χ*^2^ = 73.680, *p* < 0.001), where sufficient inter-item correlations were observed; the severe group also reached significance (*χ*^2^ = 55.910, *p* = 0.018), though with lower robustness. For depression, KMO indices ranged from 0.360 to 0.491, and Bartlett’s test was significant for the mild (*χ*^2^ = 72.812, *p* < 0.001) and severe groups (*χ*^2^ = 74.710, *p* < 0.001), but not for the moderate group (*χ*^2^ = 49.660, *p* = 0.064), suggesting a less coherent factorial structure in the latter. Although this subgroup approach aimed to observe and describe whether different consumption patterns emerge at different levels of mental health severity, the small sample size in each category limited the statistical robustness of the analysis. Therefore, these findings should be considered preliminary and interpreted with caution.

**Figure 3:**
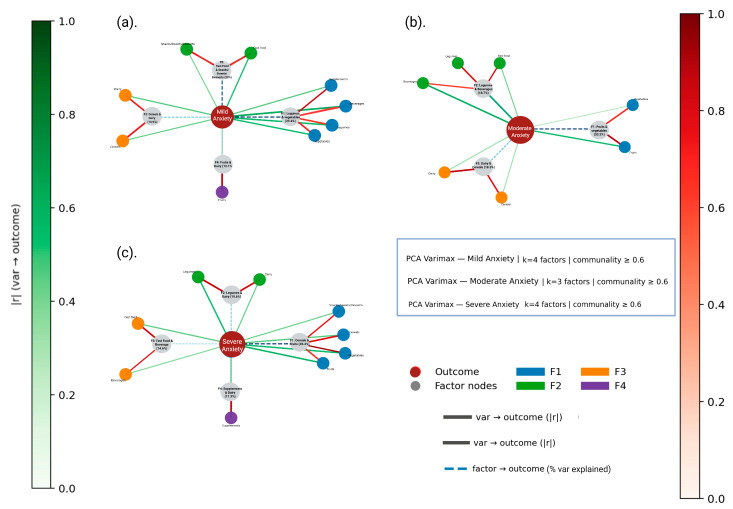
PCA–Varimax network graphs for mild (**a**), moderate (**b**) and severe (**c**) anxiety. Nodes represent food groups (blue), latent dietary factors (gray) and the anxiety subgroup outcome (red). Green edges indicate direct associations between food groups and the outcome (|*r*|), red edges show loadings from food groups to factors (|λ|^2^) and dashed blue edges represent outcome–factor connections weighted by explained variance.

**Figure 4:**
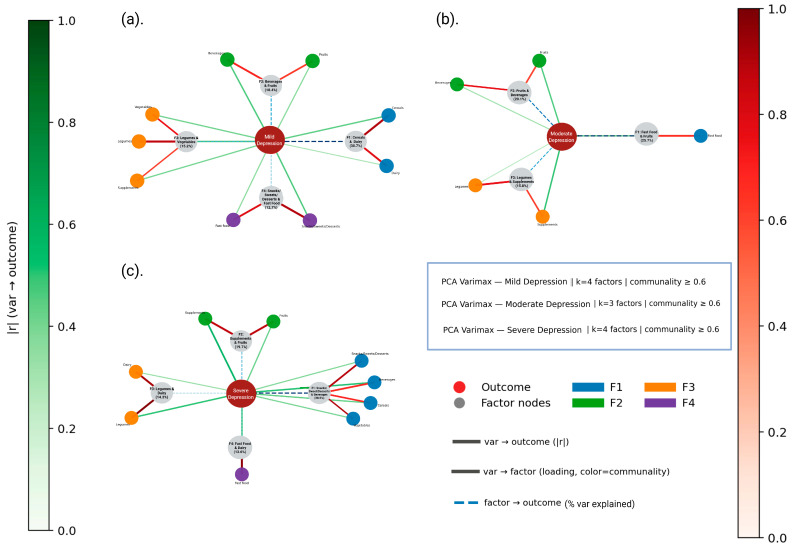
PCA–Varimax network graphs for mild (**a**), moderate (**b**) and severe (**c**) depression. Nodes represent food groups (blue), latent dietary factors (gray) and the depression subgroup outcome (red). Green edges indicate direct associations between food groups and the outcome (|*r*|), red edges show the loadings from food groups to factors (|λ|^2^) and dashed blue edges represent outcome–factor connections weighted by explained variance.

The communalities, which estimate the variance of each variable explained by the factors in the factor solution, are shown in Figure 3 and Figure 4, and the explained variance ratios (the proportion of variance attributed to each of the selected components) are presented in Tables S10–S12 of the Supplementary Materials. Among participants with mild or moderate anxiety, the primary dietary components were characterized by higher intake of healthier foods such as fruits, vegetables and pulses, suggesting a relatively balanced or health-conscious approach to eating. In these groups, the first two components explained the largest proportion of the variance (F1–F2: 48.4%; F1: 33.2%). In contrast, among participants with severe anxiety, a single component (F1) explained 35.4% of the variance and was primarily composed of snacks, sweets and desserts, sugary cereals and vegetables. The inclusion of vegetables alongside ultra-processed foods was observed to result in a less cohesive and more heterogeneous dietary profile. This pattern describes that as the severity of anxiety increases, healthy eating habits may decrease or be diluted by greater consumption of ultra-processed products. The presence of vegetables in this context is observed as irregular or compensatory eating behaviors, rather than structured dietary habits (Figure 3).

In the same sense, distinct dietary patterns were observed for depression depending on symptom severity. In cases of mild depression, one factor (F1) accounted for 30.7% of the variance and consisted mainly of cereals and dairy products. By contrast, in cases of severe depression, F1 explained 38.5% of the variance and was dominated by ultra-processed foods such as snacks, sweets, desserts, high-fat cereals and sugar-sweetened beverages, as well as vegetables. This combination shows an unstructured or compensatory eating pattern, in which vegetables are consumed alongside less healthy foods. In cases of moderate depression, two primary factors (F1 and F2) jointly explained 45.8% of the variance. F1 was mainly composed of fast food items, while F2 was associated with fruit-based beverages (Figure 4). These components describe a growing prevalence of ultra-processed food consumption, which could be associated with increasing levels of depressive symptoms.

Finally, the stress variable was also included in the data modeling because a continuous or chronic stressful environment can act as a trigger or exacerbate factors for anxiety and depression [3,33,45]. The results are shown in Figure S1 of the Supplementary Materials. For moderate stress, three components were identified (F1: Fast food + legumes; F2: Dairy products + cereals; F3: Fruits + supplements), supported by an acceptable KMO value (0.60) and a significant Bartlett’s test (*χ*^2^ = 66.5, *p* = 0.001); data are shown in Table S14 of the Supplementary Materials. Most of its variance is explained by F1 (36.7%), and it is important to note that the highest correlation for this component was with fast food. In the case of severe stress, three components also emerged (F1: Fruits + vegetables; F2: Dairy + legumes; F3: Fast food + snacks, sweets and desserts), but the statistical adequacy was lower (KMO = 0.61; Bartlett’s *χ*^2^ = 44.7, *p* = 0.152). These observed findings described that moderate stress could be associated with identifiable dietary patterns, while severe or chronic stress could be linked to disorganized or erratic eating behaviors, which could make consistent consumption profiles more difficult to discern.

### 3.7. Study Power Calculation

Power analysis of the study was conducted using simulations based on the observed frequency of depression (22%) and anxiety (30%), as well as the distribution of key dietary predictors (e.g., 33% reported consuming cake or pie on 2 or more days per week). These analyses indicated that with n=67, the power to detect ORs of 1.5 and 2.0 for logistic regression models was approximately 10% and 22%, respectively. Even for the observed effect size of cake or pie consumption (OR≈2.1), statistical power remained low (approximately 25%). Only large effect sizes (OR≥3 or 4) could be detected with moderate power (≥50%), and the effects typically reported in large epidemiological studies (OR 1.3–1.5) were below the detectable range for the present sample.

## 4. Discussion

Emotional states influence health and increase vulnerability to disease. Medical students are especially prone to mental health issues that impact well-being and performance [4,14,45]. Nutrition and bioactive compounds stand out as potential treatment options, as diet plays an important role in biological processes, prevention and the management of mental health [6,13,46]. This study identified a high frequency of anxiety (61.2%) and depression (64.2%) among medical students. These findings are consistent with previous reports in comparable populations, where between 49% and 58% of medical students present depressive symptoms, and up to 66.1% report anxiety, as shown in studies conducted in Mexico and Iran [33,45]. Although the present sample is relatively small and localized, the observed prevalence aligns with these international trends, suggesting that our cohort reflects the broader mental health burden observed in medical students. It is important to note the potential for self-selection bias, as participation was voluntary and may have attracted individuals with higher emotional distress. Nevertheless, the concordance with previous research supports the validity of the results. These findings underscore the urgent need for early detection and preventive interventions in this population, given that over half of participants presented clinically relevant symptomatology.

Higher consumption of fruits, vegetables, natural juices and dietary supplements was observed primarily among students without symptoms of anxiety or depression. This finding is similar to previous research indicating that a diet rich in fruits, vegetables and legumes is associated with lower levels of anxiety and depression [13,20,21,47]. While causality cannot be inferred, these observed associations could be seen as an exploration of whether healthier eating patterns might coexist with better mental health outcomes. In our study, food consumption revealed substantial differences in weekly food consumption associated with anxiety and depression, with d values ranging from moderate to very large (*d* ≈ 0.40 to 1.56). Students with emotional symptoms consistently showed lower intake of unprocessed foods, most notably leafy greens (*d* = –1.56) and other vegetables, while presenting higher consumption of ultra-processed foods and sweetened beverages (*d* values ≈ 0.40–0.76). These patterns mirror evidence linking psychological distress to reduced diet quality and greater reliance on energy-dense, convenience foods. The magnitude of these differences suggests that emotional symptoms may meaningfully shape dietary choices, reinforcing the relevance of assessing eating behaviors within mental health interventions targeted at university populations.

Correlation analyses revealed that emotional and contextual dynamics may significantly contribute to dietary and substance use patterns. Fast food consumption was positively associated with depression (r=0.279, p =0.022), consistent with previous studies linking ultra-processed foods to observed increases in depressive and anxiety symptoms [8,16]. Conversely, higher consumption of fruits, vegetables and legumes was associated with “pleasant moments” (p<0.05) contributing to emotionally positive experiences reported during substance use episodes, as assessed via the RASQ scale. This finding aligns with existing evidence suggesting a potential protective role of plant-rich diets in emotional well-being and neurotransmitter regulation [13,23,26]. However, the directionality of this association remains uncertain, as individuals in a more positive emotional state may also be more inclined to choose healthier foods. Further research is needed to clarify the causal pathways involved.

Dairy products were observed to show no clear association with emotional variables, exhibiting weak or negative correlations with pleasant and unpleasant emotions, which could indicate a neutral dietary pattern in terms of emotional context. In contrast, the consumption of hamburgers and soft drinks was positively and significantly correlated (p<0.05) with self-reported physical discomfort, perceived social pressure and elevated scores on psychosocial risk factors (measured through the DUSI); this might suggest that ultra-processed foods choices could be driven more by emotional or social influences than by physiological hunger. These observations are consistent with previous studies indicating that diets high in sugars and saturated fats are associated with higher levels of stress, anxiety and depression. Additionally, previous studies have reported that diets high in sugars and saturated fats are associated with greater stress, anxiety and depression. The use of tranquilizers, hallucinogens and over-the-counter medications was also significantly correlated (*p* < 0.05) with both pleasant and unpleasant emotional states, as well as with physical discomfort. These observed associations support the interpretation that some students may resort to the use of psychoactive substances as an emotional coping strategy [48], highlighting the broader psychosocial context in which eating and behavioral patterns could develop. These observations regarding diet, psychoactive substances and emotional states highlight the importance of designing larger-scale, comprehensive interventions that not only promote healthy eating habits but also examine the emotional and social factors surrounding eating behavior in university students.

It is important to note, that because this was an exploratory study, potential confounders such as sex, age, physical activity and substance use did not differ between groups and showed no detectable association with the outcomes in the bivariate screening stage. For this reason, these variables did not meet the criteria to function as confounders within this dataset, and adjusting for them would not have altered model estimates meaningfully. Nonetheless, to strengthen analytical rigor, we introduced logistic regression models that provide adjusted estimations for the main dietary exposures while reducing the number of comparisons originally explored. This approach allowed us to retain model stability given the sample size constraints while ensuring that the primary associations of interest, particularly the contrast between minimally processed and ultra-processed foods, were evaluated with appropriate statistical control.

Exploratory factor analyses and ordinal logistic regression models revealed distinct dietary patterns associated with the severity of anxiety, depression and stress (Supplementary Material, Figure S1) among students. In all models, three to four dietary components were extracted, explaining approximately 60% to 86% of the total variance. We observed significant heterogeneity in dietary patterns according to clinical category. For anxiety, a mixed pattern was observed, combining unprocessed foods (fruits, vegetables and legumes) with ultra-processed foods. The former predominated in mild and moderate cases, while the latter, especially cereals with added fats and sugars, snacks, sweets and desserts, were more prevalent in severe anxiety (F1: 35.4% of the variance).

In depression, consistent patterns were observed towards the consumption of ultra-processed foods, particularly cereals with added fats and sugars, beverages, snacks, sweets and desserts; these were observed mainly in severe depression (F1: 38.5%), in accordance with evidence linking a Western diet as factors associated with depressive symptoms [8,45,49]. Finally, a greater dispersion of patterns was observed in stress with mixed patterns; moderate stress showed fast food as the main component, with the highest component value of 0.857 (Table S12 of the Supplementary Materials) and variance (F1: 36.7%). We included stress for exploratory purposes because previous studies have shown [3,16,30,49] that stress can exacerbate emotional symptoms and lead to less healthy food choices, which could suggest a bidirectional feedback loop between mental health and diet quality. This is because the body, under constant stress, remains alert and in a state of heightened alertness, disrupting the nervous system and affecting both mental and physical health [6,22].

The ordinal logistic regression model helped us explore the dietary factors with the greatest impact on the presence of anxiety and depression. We observed that a higher consumption of cereals with added fats and sugars increased the risk of depressive symptoms by 7.4 times (95% CI: 1.19–12.15). Ultra-processed foods, high in fats, sugars, sodium and additives such as emulsifiers and artificial sweeteners, have been linked to pathophysiological changes that affect mental health [16,49]. Their effects include glucose intolerance, systemic inflammation, oxidative stress, neuroinflammation and neuronal mitochondrial dysfunction, as well as alterations in tryptophan metabolism and communication within the gut–brain axis [12,16,22]. This pro-inflammatory and neurotoxic profile could explain the observed relationship between diets of poor nutritional quality and symptoms of anxiety and depression. Furthermore, insufficient fruit and vegetable intake is a growing public health problem, particularly in university settings characterized by stress and poor eating habits. Therefore, is important to implement preventive and therapeutic strategies that promote a balanced diet to improve students’ emotional well-being, academic success and overall quality of life.

### Limitations of This Study

It is important to note some limitations of this study. First, it used data from a previous study on risk factors for suicidal behavior. Therefore, anthropometric measurements such as weight, Body Mass Index (BMI), fat percentage and other variables that could reveal stronger relationships between diet and the population’s mental health were not available. Second, in the FFQ administered to students, we only considered some foods from the dairy category; we excluded the following sections: meats, sausages, eggs, fish and seafood, corn products (Mexican snacks), cream soups and pasta and miscellaneous items. Tortillas were categorized in the cereal group, and only a few examples were included in the supplement consumption group. These adjustments, along with the elimination of certain groups and foods, were considered when evaluating snacks, foods and nutrients intended for the culturally appropriate diet. Consequently, we could not compare the remaining food groups that are an essential part of the Mexican diet. Third, the relatively small sample size (n=67) may have reduced statistical power and limited the generalizability of the findings. Consequently, non-significant findings should not be interpreted as evidence of no association but rather as a reflection of insufficient statistical power (therefore, subgroup analyses should be interpreted with caution). Future studies with substantially larger sample sizes (*n* ≥ 250–300) are necessary to reliably detect moderate associations and model multiple UPF categories simultaneously with adequate precision. Fourth, the cross-sectional design of the study precludes causal inferences; although dietary patterns were associated with mental health outcomes, it remains unclear whether diet influences emotional states or vice versa. Fifth, the self-reported nature of dietary intake may be subject to recall bias or social desirability effects, which could have influenced participants’ responses to the FFQ and substance use questionnaires. Finally, although a single continuous UPF metric (e.g., % NOVA 4) is commonly used in analytical epidemiology, it would have obscured the coexistence of both beneficial and detrimental dietary components observed in this sample. Given the exploratory objectives and the need to detect pattern-level distinctions relevant to future interventions, disaggregating exposure provided a more informative representation of dietary behaviors.

## 5. Conclusions

This exploratory cross-sectional study showed that anxiety and depression in medical students were closely aligned with distinct dietary patterns. Large effect-size differences revealed substantially lower intake of unprocessed foods—including leafy greens (*d* = −1.56), apples or pears (*d* = −1.26) and tomatoes (*d* = −1.07)—and higher intake of ultra-processed items such as pizza (*d* = 0.66), sweetened beverages (*d* = 0.66) and cereals (*d* = 0.51–0.76) among students with emotional symptoms. High consumption of sugary, high-fat cereals (>3 servings/day) was a particularly strong predictor of depressive symptom severity (OR = 7.40; 95% CI: 1.19–12.15), and the ordinal model explained a meaningful proportion of depression variance (Nagelkerke R^2^ = 0.47). Factor analyses further supported coherent symptom dimensions. Substance-use patterns, particularly moderate alcohol risk and cannabis use, co-occurred with these dietary behaviors, suggesting shared emotional or social drivers that reinforce “recreational” consumption profiles. Overall, the convergence of effect-size, regression and factorial findings suggests that dietary intake patterns operate as behavioral markers of psychological distress in this population, indicating the potential value of integrating nutritional assessment into mental health monitoring and prevention strategies in medical education settings. The observed associations should be interpreted with caution due to several limitations, including the small sample size, the cross-sectional design, potential selection bias and reliance on self-reported instruments. Future studies with substantially larger samples are needed to reliably detect moderate associations and model multiple UPF categories simultaneously with adequate precision.

## Figures and Tables

**Table 1:** General characteristics, substance consumption and substance use situations and their relationship with the presence or absence of anxiety and depression.

Variable	Total (*n* = 67)	Anxiety		*p*-Value	Depression		*p*-Value
		Absence (*n* = 26)	Presence (*n* = 41)		Absence (*n* = 24)	Presence (*n* = 43)	
**Age**	20.9 ± 1.6	20.9 ± 1.7	20.8 ± 1.4	0.681	20.9 ± 1.6	20.9 ± 1.4	0.998
**Sex**							
Female	40 (59.7)	19 (73.1)	21 (51.2)	0.075	15 (62.5)	24 (57.1)	0.648
Male	27 (40.3)	7 (26.9)	20 (48.8)		9 (37.5)	18 (42.9)	
**Foreigner**							
Yes	32 (47.8)	9 (34.6)	23 (56.1)	0.086	11 (45.8)	21 (47.6)	0.568
No	35 (52.2)	17 (65.4)	18 (43.9)		13 (54.2)	22 (52.4)	
**Working**							
Yes	11 (16.4)	3 (11.5)	8 (19.5)	0.391	2 (8.3)	9 (21.4)	0.349
No	56 (83.6)	23 (88.5)	33 (80.5)		22 (91.7)	34 (78.6)	
**Physical activity**							
Low	18 (26.9)	4 (15.4)	14 (34.1)	0.234	2 (8.3)	16 (35.7)	0.071
Medium	17 (25.4)	8 (30.8)	9 (22)		8 (33.3)	9 (21.4)	
High	32 (47.7)	14 (53.8)	18 (43.9)		14 (58.3)	18 (42.9)	
**Alcohol**							
Low risk	54 (80.6)	22 (84.6)	32 (78)	0.647	23 (95.8)	31 (73.8)	0.051
Medium risk	12 (17.9)	4 (15.4)	8 (19.5)		1 (4.2)	11 (23.8)	
High risk	1 (1.5)	0 (0)	1 (2.4)		0 (0)	1 (2.4)	
Probable Addiction	0 (0)	0 (0)	0 (0)		0 (0)	0 (0)	
**Cannabis**							
No symptoms of addiction	61 (91)	24 (92.3)	37 (90.2)	0.805	22 (87.5)	40 (92.9)	0.959
Problematic use	4 (6)	1 (3.8)	3 (7.3)		2 (8.3)	2 (4.8)	
Symptoms of addiction	2 (3)	1 (3.8)	1 (2.4)		0 (0)	1 (2.4)	

Comparisons between groups were obtained using Chi-square test.

**Table 2:** Frequency of food consumption and its relationship with the presence or absence of anxiety and depression symptoms.

Variable	Food	Total (*n* = 67)	Anxiety		*p*-Value	Depression		***p-*** **Value**
			Absence (*n* = 26)	Presence (*n* = 41)		Absence (*n* = 24)	**Presence (*n* = 43)**	
**A. Consumption of unprocessed or minimally processed foods**	Apple or pear	4.00 ± 2.188	4.77 ± 2.250	3.51 ± 2.026	**0.012**	4.00 ± 2.085	4.29 ± 2.225	0.318
	Guava	2.30 ± 1.723	2.81 ± 2.136	1.98 ± 1.332	**0.042**	2.38 ± 1.861	2.29 ± 1.782	0.431
	Red tomato	4.81 ± 2.091	5.46 ± 2.502	4.39 ± 1.686	**0.02**	5.04 ± 2.368	5.07 ± 2.035	0.481
	Leaved greens (chard, spinach, quelites)	3.78 ± 2.131	4.73 ± 2.491	3.17 ± 1.626	**0.001**	4.75 ± 2.270	3.32 ± 2.091	**0.012**
	Carrot	2.85 ± 2.258	3.65 ± 2.667	2.34 ± 1.811	**0.010**	3.21 ± 2.536	2.75 ± 2.119	0.244
	Cucumber	3.16 ± 2.027	3.73 ± 2.108	2.80 ± 1.913	**0.034**	3.46 ± 1.978	2.96 ± 2.009	0.189
	Onion	4.52 ± 2.427	5.19 ± 2.593	4.10 ± 2.245	**0.041**	4.13 ± 2.576	5.04 ± 2.442	0.1
	Lentils	1.94 ± 1.301	1.54 ± 0.948	2.20 ± 1.436	**0.022**	1.75 ± 1.294	2.04 ± 1.290	0.215
	Natural juice without added sugar	1.49 ± 1.050	1.23 ± 0.710	1.66 ± 1.196	**0.035**	1.38 ± 0.824	1.57 ± 1.200	0.245
**B. Consumption of ultra-processed foods**	Pizza	1.87 ± 1.100	1.46 ± 0.761	2.12 ± 1.208	**0.004**	1.71 ± 0.806	1.79 ± 1.287	0.397
	Hot dogs	1.36 ± 0.667	1.19 ± 0.402	1.46 ± 0.778	**0.033**	1.29 ± 0.464	1.39 ± 0.875	0.397
	Times a day eaten (0 to 6 times) fast-food	2.39 ± 1.058	1.96 ± 0.720	2.66 ± 1.153	**0.002**	2.17 ± 1.049	2.21 ± 0.630	0.424
	Bakery donuts and churros	1.73 ± 0.978	1.46 ± 0.706	1.90 ± 1.091	**0.024**	1.63 ± 0.770	1.64 ± 0.731	0.466
	Boxed cereal with sweetened corn flakes	1.81 ± 1.417	1.46 ± 1.140	2.02 ± 1.541	**0.046**	1.54 ± 0.977	1.89 ± 1.449	0.153
	Cornflake cereal	1.93 ± 1.480	2.12 ± 1.646	1.62 ± 1.134	0.071	1.46 ± 0.658	2.14 ± 1.557	**0.020**
	Cake or pie	1.51 ± 0.975	1.27 ± 0.533	1.66 ± 1.153	**0.033**	1.29 ± 0.550	1.39 ± 0.737	0.287
	Caramel popcorn	1.12 ± 0.327	1.04 ± 0.196	1.17 ± 0.381	**0.033**	1.08 ± 0.282	1.11 ± 0.315	0.388
	Sweet cookies of all kinds	2.09 ± 1.485	2.24 ± 1.640	1.85 ± 1.190	0.128	1.79 ± 1.062	2.36 ± 1.367	**0.050**
	Natural drinking yogurt	1.46 ± 0.959	1.19 ± 0.402	1.63 ± 1.157	**0.015**	1.29 ± 0.690	1.43 ± 0.742	0.247
	Regular soda	2.07 ± 1.418	1.69 ± 0.928	2.32 ± 1.619	**0.025**	1.83 ± 1.523	2.11 ± 0.916	0.223
	Natural juice with added sugar	1.67 ± 1.147	1.38 ± 0.898	1.85 ± 1.256	**0.040**	1.42 ± 0.830	1.71 ± 1.272	0.158
	Industrially processed beverages or flavored water with sugar (tea drinks, fruit drinks, etc.)	1.43 ± 1.033	1.00 ± 0.0	1.66 ± 1.153	**<0.001**	1.08 ± 0.408	1.43 ± 0.836	**0.030**
	Flavored juices nectars with added sugar	1.31 ± 0.925	1.08 ± 0.392	1.46 ± 1.120	**0.024**	1.42 ± 0.830	1.71 ± 1.272	0.088
	Beverages consumed per day (0 to 6)	5.31 ± 1.725	5.27 ± 1.564	4.37 ± 1.757	**0.016**	4.92 ± 1.909	4.75 ± 1.669	0.371
**C. Supplement consumption**	Days (0 to 7) of other supplements consumed	1.43 ± 1.569	1.00 ± 0.0	1.71 ± 1.965	**0.013**	1.04 ± 0.204	1.71 ± 2.016	**0.045**

Statistical analysis was performed using MANOVA. Sections A, B and C correspond to consumption (days/week) of unprocessed or minimally processed foods, consumption (days/week) of ultra-processed foods and supplement consumption (days/week). *p* < 0.05 are highlighted in bold.

**Table 3:** Effect size estimates for weekly food consumption differences by anxiety and depression symptoms.

Variable	Food	Anxiety (Absence/Presence)	
		Cohen’s *d*	95% CI
** A. Consumption of unprocessed or minimally processed foods **	Apple or pear	−1.257	−2.35–−0.17
	Guava	−0.832	−1.78–0.12
	Red tomato	−1.071	−2.2–−0.06
	Leaved greens chard, spinach, quelites	−1.560	−2.67–−0.45
	Carrot	−1.312	−2.52–−0.11
	Cucumber	−0.926	−1.95–0.1
	Onion	−1.095	−2.34–0.15
	Lentils	0.657	0.08–1.24
	Natural juice without added sugar	0.428	−0.04–0.89
** B. Consumption of ultra-processed foods **	Pizza	0.66	0.18–1.14
	Hot dogs	0.271	−0.18–0.56
	Bakery doughnuts and churros	0.441	0.002–0.88
	Boxed cereal with sweetened corn flakes	0.563	−0.09–1.22
	Cornflake cereal	0.507	−0.17–1.19
	Cake or pie	0.389	−0.03–0.81
	Caramel popcorn	0.132	−0.009–0.27
	Sweet cookies of all kinds	0.398	−0.29–1.09
	Natural drinking yogurt	0.442	0.05–0.84
	Regular soda	0.625	0.002–1.25
	Natural juice with added sugar	0.469	−0.06–1.0
	Industrially processed beverages or flavored water with sugar (tea drinks, fruit drinks, etc.)	0.659	0.003–0.77
	Flavored juices nectars with added sugar	0.386	0.003–0.77
** C. Supplement consumption **	Days (0 to 7) of other supplements consumed	0.707	0.09–1.33
**Variable**	**Food**	**Depression (Absence/Presence)**	
		**Cohen’s *d***	**95% CI**
** A. Consumption of unprocessed or minimally processed foods **	Leaved greens (chard, spinach, quelites)	−1.5212	−2.62–−0.407
** B. Consumption of ultra-processed foods **	Cornflake cereal	0.756	0.15–1.36
	Sweet cookies of all kinds	0.494	−0.18–1.17
	Industrially processed beverages or flavored water with sugar (tea drinks, fruit drinks, etc.)	0.464	0.08–0.85
** C. Supplement consumption **	Days (0 to 7) of other supplements consumed	0.625	0.13–1.24

Cohen’s d represents the standardized mean difference in weekly food consumption between students with and without anxiety or depression. Negative values indicate lower consumption among students with symptoms, and positive values indicate higher consumption.

**Table 4:** Logistic regression analysis of weekly food consumption frequency and daily consumption of food groups that predict symptoms of anxiety and depression.

Food *	Anxiety		Depression	
	β	*p*-Value	β	*p*-Value
Leaved greens (chard, spinach, quelites)	−0.138	0.552	−0.283	**0.030**
Food group **				
Legumes	0.402	**0.002**	0.140	0.388

* Number of times the food is consumed per week. ** Number of times that food group is consumed per day (0–6 times). *p* < 0.05 is highlighted in bold. The table only shows significant values.

**Table 5:** Ordinal logistic regression for depression and anxiety severity.

Variable/Category	*χ^2^* (Δ*χ*^2^)	df	*p*	Nagelkerke R^2^	OR	95% CI (OR)
Depression model	42.9	17	**<0.001**	0.47	-	-
Cereals high in fat/sugar (>3 portions/day)	-	-	-	-	7.40	1.19–12.154
Legumes (2–3 portions/day)	-	-	-	-	0.26	−1.28–8.587
Anxiety model	23.6	16	0.10	0.29	-	-
Cereals high in fat/sugar (>3 portions/day)	-	-	-	-	7.40	0.339–8.308

Variables were expressed as servings/day of eight food groups included in the consumption frequency (dairy, fruits, vegetables, legumes, cereals, fast food, beverages, snacks/sweets/desserts). Abbreviations: Delta Chi-square ( $\chi^{2},\Delta \chi^{2}$), degrees of freedom (df), *p*-value (*p*), Odds Ratio (OR) and confidence intervals (CIs). Depression categories: absence/mild/moderate/severe. *p*-values < 0.05 are highlighted in bold.

## Data Availability

The data that support the findings of this study are available from the corresponding author upon reasonable request.

## Supplementary Materials

The following supporting information can be downloaded at https://www.mdpi.com/article/10.3390/nu18010104/s1, Table S1: Aiken’s V index of validity, reproducibility and homogeneity of the Consumption Frequency questionnaire; Table S2. Content Validity Ratio Lawshe Method of the Consumption Frequency Questionnaire; Table S3. Results of the internal validity and reliability of the Consumption Frequency Questionnaire; Table S4. Internal validation and reliability of the Consumption Frequency Questionnaire; Table S5. Substance use situations and their relationship with anxiety and depression; Table S6. Frequency of consumption by portion (eaten per day) of food groups and its relationship with anxiety and depression symptoms; Table S7. Logistic regression analysis of weekly food consumption frequency predicting anxiety and depression symptoms; Table S8. Logistic regression analysis of anxiety and depression symptoms by number of daily servings consumed from food groups; Table S9. Logistic regression analysis of anxiety/depression symptoms based on daily consumption frequency of food groups; Table S10. Component matrix to severity level in anxiety; Table S11. Component matrix to severity level in depression; Table S12. Component matrix to severity level in stress; Table S13. Kaiser–Meyer–Olkin (KMO) measure of sampling adequacy and Bartlett’s test of sphericity for food group data across anxiety and depression levels; Table S14. Kaiser–Meyer–Olkin (KMO) measure of sampling adequacy and Bartlett’s test of sphericity for food group data across stress levels; Figure S1. PCA–Varimax network graphs for mild (a), moderate (b) and severe (c) stress.

## Abbreviations

The following abbreviations are used in this manuscript:

FFQ	Food Frequency Questionnaire
PCA	Principal Component Analysis
ADHD	Attention Deficit Hyperactivity Disorder
PAHO	Pan American Health Organization
WHO	World Health Organization
INEGI	National Institute of Statistics and Geography
ENSANUT	National Health and Nutrition Survey
DASS-21	Depression, Anxiety and Stress Scale
IPAQ	International Physical Activity Questionnaire
AUDIT	Alcohol Use Disorders Identification Test
CAST	Cannabis Addiction Test
DUSI	Drug Use Situations Inventory
RASQ	Reasons for Attempting Suicide Questionnaire
CVR	Content Validity Ratio
MANOVA	Multivariate Analysis of Variance
SD	Standard Deviation
KMO	Kaiser–Meyer–Olkin

## References

[1] Kris-Etherton P.M., Petersen K.S., Hibbeln J.R., Hurley D., Kolick V., Peoples S., Rodriguez N., Woodward-Lopez G. (2021). Nutrition and behavioral health disorders: Depression and anxiety. Nutr. Rev..

[2] Steiner-Hofbauer V., Holzinger A. (2020). How to Cope with the Challenges of Medical Education? Stress. Depression, and Coping in Undergraduate Medical Students. Acad. Psychiatry.

[3] Avila-Carrasco L., Díaz-Avila D.L., Reyes-López A., Monarrez-Espino J., Garza-Veloz I., Velasco-Elizondo P., Vázquez-Reyes S., Mauricio-González A., Solís-Galván J.A., Martinez-Fierro M.L. (2022). Anxiety, depression, and academic stress among medical students during the COVID-19 pandemic. Front. Psychol..

[4] Martinez-Fierro M.L., Reyes-Hurtado J.R., Ayala-Haro A.E., Avila-Carrasco L., Ramirez-Hernandez L.A., Lozano-Razo G., Zavala-Rayas J., Vazquez-Reyes S., Mauricio-Gonzalez A., Velasco-Elizondo P. (2025). The hidden risk factors behind of suicidal behavior in medical students: A cross-sectional cohort study in Mexico. Front. Psychiatry.

[5] Pan American Health Organization (2023). A New Agenda for Mental Health in the Americas: Report of the Pan American Health Organization High-Level Commission on Mental Health and COVID-19.

[6] Giacobbe J., Benoiton B., Zunszain P., Pariante C.M., Borsini A. (2020). The Anti-Inflammatory Role of Omega-3 Polyunsaturated Fatty Acids Metabolites in Pre-Clinical Models of Psychiatric, Neurodegenerative, and Neurological Disorders. Front. Psychiatry.

[7] Gibson-Smith D., Bot M., Brouwer I.A., Visser M., Giltay E.J., Penninx B. (2020). Association of food groups with depression and anxiety disorders. Eur. J. Nutr..

[8] Hecht E.M., Rabil A., Steele E.M., Abrams G.A., Ware D., Landy D.C., Hennekens C.H. (2022). Cross-sectional examination of ultra-processed food consumption and adverse mental health symptoms. Public Health Nutr..

[9] Interián-Gómez L., Aguila-Gutiérrez S.E., Esquivias-López K.M., Pulido-De la Cruz V.A., Silva-Arzola N.J., Gonzalez-Becerra K. (2021). Componentes alimenticios, estado de ánimo y su relación con el sistema inmune en COVID-19. RESPYN Rev. Salud Pública Nutr..

[10] Kalin N.H. (2020). The Critical Relationship Between Anxiety and Depression. Am. J. Psychiatry.

[11] Kim S., Jeong J., Kang J., Kim J., Yang Y.J. (2024). A comparative study on eating habits and mental health of Korean middle school students according to their bedtime across regions: Using data from the 2020–2022 Korea Youth Risk Behavior Survey. Nutr. Res. Pract..

[12] Liu C.H., Zhang G.Z., Li B., Li M., Woelfer M., Walter M., Wang L. (2019). Role of inflammation in depression relapse. J. Neuroinflamm..

[13] Liu T., Ma Y., Zhang R., Zhong H., Wang L., Zhao J., Yang L., Fan X. (2019). Resveratrol ameliorates estrogen deficiency-induced depression- and anxiety-like behaviors and hippocampal inflammation in mice. Psychopharmacology.

[14] Alzahrani S.H., Saeedi A.A., Baamer M.K., Shalabi A.F., Alzahrani A.M. (2020). Eating Habits Among Medical Students at King Abdulaziz University, Jeddah, Saudi Arabia. Int. J. Gen. Med..

[15] Chen L.M., Bao C.H., Wu Y., Liang S.H., Wang D., Wu L.Y., Huang Y., Liu H.R., Wu H.G. (2021). Tryptophan-kynurenine metabolism: A link between the gut and brain for depression in inflammatory bowel disease. J. Neuroinflamm..

[16] Coletro H.N., Mendonça R.D., Meireles A.L., Machado-Coelho G.L.L., Menezes M.C. (2022). Ultra-processed and fresh food consumption and symptoms of anxiety and depression during the COVID 19 pandemic: COVID Inconfidentes. Clin. Nutr. ESPEN.

[17] Di Lorenzo C., Colombo F., Biella S., Stockley C., Restani P. (2021). Polyphenols and Human Health: The Role of Bioavailability. Nutrients.

[18] Francis H.M., Stevenson R.J., Chambers J.R., Gupta D., Newey B., Lim C.K. (2019). A brief diet intervention can reduce symptoms of depression in young adults—A randomised controlled trial. PLoS ONE.

[19] Fusar-Poli L., Gabbiadini A., Ciancio A., Vozza L., Signorelli M.S., Aguglia E. (2022). The effect of cocoa-rich products on depression. anxiety, and mood: A systematic review and meta-analysis. Crit. Rev. Food Sci. Nutr..

[20] Marx W., Lane M., Hockey M., Aslam H., Berk M., Walder K., Borsini A., Firth J., Pariante C.M., Berding K. (2021). Diet and depression: Exploring the biological mechanisms of action. Mol. Psychiatry.

[21] Marx W., Moseley G., Berk M., Jacka F. (2017). Nutritional psychiatry: The present state of the evidence. Proc. Nutr. Soc..

[22] Lane M.M., Gamage E., Travica N., Dissanayaka T., Ashtree D.N., Gauci S., Lotfaliany M., O’Neil A., Jacka F.N., Marx W. (2022). Ultra-Processed Food Consumption and Mental Health: A Systematic Review and Meta-Analysis of Observational Studies. Nutrients.

[23] Radavelli-Bagatini S., Anokye R., Bondonno N.P., Sim M., Bondonno C.P., Stanley M.J., Harms C., Woodman R., Magliano D.J., Shaw J.E. (2021). Association of habitual intake of fruits and vegetables with depressive symptoms: The AusDiab study. Eur. J. Nutr..

[24] Rossa-Roccor V., Richardson C.G., Murphy R.A., Gadermann A.M. (2021). The association between diet and mental health and wellbeing in young adults within a biopsychosocial framework. PLoS ONE.

[25] Sadeghi O., Keshteli A.H., Afshar H., Esmaillzadeh A., Adibi P. (2021). Adherence to Mediterranean dietary pattern is inversely associated with depression, anxiety and psychological distress. Nutr. Neurosci..

[26] Shamabadi A., Kafi F., Bafrani M.A., Asadigandomani H., Basti F.A., Akhondzadeh S. (2023). L-theanine adjunct to sertraline for major depressive disorder: A randomized, double-blind, placebo-controlled clinical trial. J. Affect. Disord..

[27] Tayab M.A., Islam M.N., Chowdhury K.A.A., Tasnim F.M. (2022). Targeting neuroinflammation by polyphenols: A promising therapeutic approach against inflammation-associated depression. Biomed. Pharmacother..

[28] Zheng Z.H., Tu J.L., Li X.H., Hua Q., Liu W.Z., Liu Y., Pan B.X., Hu P., Zhang W.H. (2021). Neuroinflammation induces anxiety- and depressive-like behavior by modulating neuronal plasticity in the basolateral amygdala. Brain Behav. Immun..

[29] Keck M.M., Vivier H., Cassisi J.E., Dvorak R.D., Dunn M.E., Neer S.M., Ross E.J. (2020). Examining the Role of Anxiety and Depression in Dietary Choices among College Students. Nutrients.

[30] Fayyazi E., Mohammadi E., Aghamohammadi V. (2024). Association between major dietary patterns and mental health problems among college students. J. Educ. Health Promot..

[31] Kundu S., Rejwana N., Al Banna M.H., Kawuki J., Ghosh S., Alshahrani N.Z., Dukhi N., Kundu S., Dey R., Hagan J.E. (2022). Linking Depressive and Anxiety Symptoms with Diet Quality of University Students: A Cross-Sectional Study during the COVID-19 Pandemic in India. Healthcare.

[32] Genç S., Junqueıra-goncalves M.P., Genç M., Majumdar A. (2023). Perceptions of university students on nutrition as a useful tool to manage anxiety and depression levels. Gıda Ve Yem Bilim. Teknol. Derg..

[33] Miri A., Norouzzadeh M., Mozafari F., Rajabipour E., Souri-Naseri N., Jahantigh M. (2021). Investigating the Relationship between Depression and Dietary Patterns in Students of Zabol University of Medical Sciences. J. Zabol Med. Sch..

[34] Secretaría de Salud (SSA) Food Frequency Questionnaire for Adolescents and Adults (≥12 Years of Age). ENSANUT 2021, Mexico. https://ensanut.insp.mx/encuestas/ensanutcontinua2021/descargas.php.

[35] Francisca S.P.R., Vinet Eugenia V. (2016). Use of the Depression Anxiety Stress Scales (DASS-21) as a Screening Instrument in Young People with Clinical Problems. Psychol. Res. Act.

[36] Seron P., Munoz S., Lanas F. (2010). Physical activity level measured through the international physical activity questionnaire in the Chilean population. Chil. Med. J..

[37] Alvarado M., Acuña G., Santis R., Arteaga O. (2009). Validez y confiabilidad de la versión chilena del Alcohol Use Disorders Identification Test (AUDIT). Rev. Med. Chile.

[38] Legleye S. (2018). The Cannabis Abuse Screening Test and the DSM-5 in the general population: Optimal thresholds and underlying common structure using multiple factor analysis. Int. J. Methods Psychiatr. Res..

[39] Barragán L., Flores M., Morales S., González J., Martínez M., Ayala H. (2014). Programa de Satisfactores Cotidianos para Usuarios con Dependencia a Sustancias Adictivas: Manual del Terapeuta.

[40] Holden R.R., McLeod L.D. (2000). The structure of the Reasons for Attempting Suicide Questionnaire (RASQ) in a nonclinical adult population. Personal. Individ. Differ..

[41] Martinez-Fierro M.L., Ramirez-Hernandez L.A., Trejo-Ortiz P.M., Lozano-Razo G., Zavala-Rayas J., Vazquez-Reyes S., Velasco-Elizondo P., Mauricio-Gonzalez A., Araujo-Espino R., Mollinedo-Montaño F.E. (2025). Psychosocial and Mental Health Determinants of Suicidal Behavior Among Nursing Students: A Cross-Sectional Study in Mexico. Nurs. Rep..

[42] Hermosillo-De la Torre A.E., Méndez-Sánchez C., González-Betanzos F. (2020). Evidence of factorial validity of the Beck Hopelessness Scale in Spanish with clinical and nonclinical samples. Act Colomb. Psychol..

[43] Petrus R.R., Sobral P.J.D.A., Tadini C.C., Gonçalves C.B. (2021). Technology, The NOVA classification system: A critical perspective in food science. Trends Food Sci. Technol..

[44] von Elm E., Egger M., Altman D.G., Pocock S.J., Gotzsche P.C., Vandenbroucke J.P. (2009). Declaración de la Iniciativa STROBE (Strengthening the Reporting of Observational studies in Epidemiology): Directrices para la comunicación de estudios observacionales. Nefrología.

[45] Robles-Rivera K., Limón-Rojas A.E., Wakida-Kuzunoki G.H., Moreno-Altamirano L., Vázquez-Rivera M., Romero-Romero E., Rojas-Hernández M.T., Morales-Carmona R.O. (2025). Factors associated with depression, anxiety, and stress in Mexican medical students: A cross-sectional study. Curr. Psychol..

[46] Katrenčíková B., Vaváková M., Paduchová Z., Nagyová Z., Garaiova I., Muchová J., Ďuračková Z., Trebatická J. (2021). Oxidative Stress Markers and Antioxidant Enzymes in Children and Adolescents with Depressive Disorder and Impact of Omega-3 Fatty Acids in Randomised Clinical Trial. Antioxidants.

[47] Liwinski T., Lang U.E. (2023). Folate and Its Significance in Depressive Disorders and Suicidality: A Comprehensive Narrative Review. Nutrients.

[48] Molodynski A., Thomas L., Murtaza K., Marie F.S., Maha L.C., Telma F.D.A., Rawan M., Anindya K., Umberto V., Fiona M. (2021). Cultural variations in wellbeing, burnout and substance use amongst medical students in twelve countries. Int. Rev. Psychiatry.

[49] Faisal-Cury A., Leite M.A., Escuder M.M.L., Levy R.B., Peres M.F.T. (2022). The relationship between ultra-processed food consumption and internalising symptoms among adolescents from São Paulo city. Southeast Braz. Public Health Nutr..

