# Supplementary material for: Dietary Intake Patterns, Substance Use and Their Association with Anxiety and Depression Symptoms in Medical Students in Mexico: A Cross-Sectional Study
Source: Nutrients. 2025 Dec 28;18(1):104. doi: 10.3390/nu18010104 (PMC12787937; doi:10.3390/nu18010104)
Supplement: Supplementary file 1 [file nutrients-18-00104-s001.zip › nutrients-3995955-supplementary.pdf]

Supplementary material

Table S1. Aiken's V index of validity, reproducibility and homogeneity of the Consumption Frequency questionnaire.

Validity Index	Aiken's V	Lower CI (95%)	Upper CI (95%)
**Food Consumption Frequency**	0.93	0.8	0.97
**Number of Items**		115	

The table shows the results of Aiken's V index used to assess the validity, reproducibility and homogeneity of the FFQ. This value of 0.93 indicates high agreement and content validity of the instrument. It is important to note that the questionnaire evaluated consists of a total of 115 food consumption items.

Table S2. Content Validity Ratio Lawshe Method of the Consumption Frequency Questionnaire.

Content Validity Ratio (Lawshe Method)	CVR
Writing Style	0.88
Relevance	0.89
Structure	0.90
Language	0.88
Overall CVR	0.88
Number of Elements	115

The table shows the results of the Content Validity Coefficient (CVR) obtained using the Lawshe Method to evaluate the qualitative characteristics of the Consumption Frequency Questionnaire (FFQ), which consists of 115 items.

Table S3. Results of the internal validity and reliability of the consumption frequency questionnaire.

Cronbach's alpha obtained (α)	
**Value**	**Interpretation**
0.915	Excellent
Elements	115

The sample presents the results of the instrument's validity and internal reliability.

Table S4. Internal validation and reliability of the consumption frequency questionnaire.

Validity Index	*r*	Intraclass Correlation Coefficient	95% CI
**Consumption frequency questionnaire**	0.915	0.915	0.866-0.953

The table shows the results of the internal validation and reliability of the consumption frequency questionnaire (115 items), using a sample of 30 participants (n = 30).

Table S5. Substance use situations and their relationship with anxiety and depression.

Variable	Total (*n*=67)	Anxiety		*P*-value	Depression		*P*-value
		Absence (*n*=26)	Presence (*n*=41)		Absence (*n*=24)	Presence (*n*=43)	
Pleasant emotions							
N/A	42 (62.7)	18 (69.2)	24 (58.5)		16 (66.7)	26 (61.9)	
>50%	6 (9)	3 (11.5)	3 (7.3)	0.396	2 (8.3)	4 (9.5)	0.607
<50%	19 (28.3)	5 (19.2)	14 (34.1)		6 (25)	13 (28.6)	
Unpleasant emotions							
N/A	42 (62.7)	18 (69.2)	24 (58.8)		16 (66.7)	26 (61.9)	
>50%	2 (3)	1 (3.8)	1 (2.4)	0.584	1 (4.2)	1 (2.4)	0.671
<50%	23 (34.3)	7 (26.9)	16 (39)		7 (29.2)	16 (35.7)	
Physical discomfort							
N/A	41 (61)	18 (69.2)	23 (56.1)		16 (66.7)	25 (59.5)	
>50%	26 (38.8)	8 (30.8)	18 (43.9)	0.282	0 (0)	0 (0)	0.381
<50%	0 (0)	0 (0)	0 (0)		8 (33.3)	18 (40.5)	
Self-control							
N/A	41 (61.5)	18 (69.2)	23 (56.1)		16 (66.7)	25 (59.2)	
>50%	1 (1.5)	0 (0)	1 (2.4)	0.456	0 (0)	1 (2.4)	0.644
<50%	25 (37)	8 (30.8)	17 (41.5)		8 (33.3)	17 (38.1)	
Physical need							
N/A	41 (61.2)	18 (69.2)	23 (56.1)		16 (66.7)	25 (59.5)	
>50%	0 (0)	0 (0)	0 (0)	0.282	0 (0)	0 (0)	0.381
<50%	26 (38.8)	8 (30.8)	18 (43.9)		8 (33.3)	18 (40.5)	
Pleasant moments							
N/A	41 (61.2)	18 (69.2)	23 (56.1)		16 (66.7)	25 (59.5)	
>50%	6 (9)	1 (3.8)	5 (12.2)	0.405	0 (0)	5 (11.9)	**0.011**
<50%	20 (29.8)	7 (26.9)	13 (31.7)		8 (33.3)	13 (28.6)	

Social pressure							
N/A	41 (61.2)	18 (68.2)	23 (56.1)		16 (66.7)	25 (59.5)	
>50%	1 (1.5)	0 (0)	1 (2.4)	0.456	0 (0)	1 (2.4)	0.644
<50%	25 (37.3)	8 (30.8)	17 (41.5)		8 (33.3)	17 (38.1)	
Conflicts with others							
N/A	41 (61.2)	18 (69.2)	23 (56.1)		16 (66.7)	25 (59.5)	
>50%	0 (0)	0 (0)	0 (0)	0.282	0 (0)	0 (0)	0.381
<50%	26 (38.8)	8 (30.8)	18 (43.9)		8 (33.3)	18 (40.5)	

Comparisons between groups were obtained using Chi-square test. $P < 0.05$ are highlighted in bold.

Table S6. Frequency of consumption by portion (eaten per day) of food groups and its relationship with anxiety and depression symptoms.

Variable	Food	Total (*n*=67)	Anxiety			Depression		
			Absence (*n*=26)	Presence (*n*=41)	*P*-value	Absence (*n*=24)	Presence (*n*=43)	*P*-value
**Consumption (portions/day) of food groups***	Portions of dairy products eaten or consumed per day	2.78±1.098	2.71±0.873	2.88±1.395	0.283	2.79±1.351	2.79±0.995	0.493
	Portions of fruit eaten per day	3.16±0.898	3.15±1.014	3.19±0.694	0.413	3.13±0.797	3.25±0.799	0.288
	Portions of vegetables eaten per day	3.55±1.063	3.44±1.050	3.73±1.079	0.14	3.63±1.096	3.54±0.962	0.379
	Portions of fast-food eaten per day	2.33±0.927	2.41±0.921	2.19±0.939	0.173	2.29±1.122	2.25±0.752	0.439
	Portions of legumes eaten per day	2.79±1.023	2.93±1.034	2.58±0.987	0.085	2.67±1.129	2.79±0.833	0.336
	Portions of cereals eaten per day	2.63±0.885	2.59±0.774	2.69±1.050	0.328	2.71±0.955	2.50±0.793	0.201
	Portions of beverages eaten per day	4.72±1.730	4.37±1.757	5.27±1.564	**0.016**	4.92±1.909	4.75±1.669	0.371
	Portions of snacks, sweets, and desserts eaten per day	2.79±1.108	2.83±1.093	2.73±1.151	0.365	2.67±1.167	2.86±1.008	0.535
	Portions of supplements eaten or consumed per day	1.66±1.213	1.73±1.205	1.54±1.240	0.266	1.58±1.349	1.50±0.793	0.396

MANOVAS statistical analysis was performed. *Corresponds to the consumption (portions/day) of food groups. $P < 0.05$ are highlighted in bold.

Table S7. Logistic regression analysis of weekly food consumption frequency predicting anxiety and depression symptoms.

Food*	Anxiety		Depression	
	β	*P*-Value	β	*P*-Value
Apple or pear	-0.024	0.921	-	-
Guava	-0.193	0.308	-	-
Red tomato	-0.020	0.935	-	-
Leaved greens (chard, spinach, quelites)	-0.138	0.552	-0.283	**0.030**
Carrot	-0.254	0.170	-	-
Cucumber	-0.206	0.369	-	-
Onion	0.322	0.087	-	-
Lentils	0.253	0.324	-	-
Natural juice without added sugar	0.042	0.831	-	-
Hot dog	0.010	0.961	-	-
Bakery donuts and churros	0.027	0.883	-	-
Boxed cereal with sweetened corn flakes	0.119	0.547	-	-
Corn flakes cereal	-0.173	0.538	0.068	0.739
Cake or pie	0.290	0.250	-	-
Caramel popcorn	-0.068	0.744	-	-
Sweet cookies of all kinds	0.009	0.964	0.098	0.359
Natural drinking yogurt	-0.095	0.719	-	-
Regular soda	0.091	0.683	-	-
Natural juice with added sugar	0.006	0.973	-	-
Industrially processed beverages or flavored water with sugar (tea drinks, fruit drinks, etc.)	0.394	0.073	0.198	0.357
Flavored juices nectars with added sugar	-0.056	0.811	-	-
Other supplements consumption	0.192	0.185	0.137	0.186

* Number of times the food is consumed per week. $P < 0.05$ are highlighted in bold

Table S8. Logistic regression analysis of anxiety and depression symptoms by number of daily servings consumed from food groups.

Food group*	Anxiety		Depression	
	β	*P*-Value	β	*P*-Value
Fruts	0.094	0.478	0.263	0.166
Vegetables	-0.235	0.141	-0.240	0.158
Legumes	-0.009	0.954	0.178	0.233
Drinks	0.246	0.108	-0.041	0.741
Fast food	-0.082	0.592	-0.244	0.225
Cereals	-0.198	0.131	0.061	0.723
Snacks, sweets, and desserts	0.142	0.417	0.371	0.088

* Number of daily servings consumed from that food group.

Table S9. Logistic regression analysis of anxiety/depression symptoms based on daily consumption frequency of food groups.

Food group*	Anxiety		Depression	
	β	*P*-Value	β	*P*-Value
Fruts	-0.015	0.923	-0.001	0.994
Vegetables	-0.170	0.306	-0.038	0.840
Legumes	0.402	**0.002**	0.140	0.388
Drinks	0.064	0.676	0.133	0.441
Fast food	0.006	0.962	-0.074	0.622
Cereals	0.016	0.908	0.100	0.483
Snacks, sweets, and desserts	-0.137	0.380	0.049	0.770

* Number of times per day that food group was consumed (0–6 times). $P < 0.05$ are highlighted in bold

Table S10. Component matrix to severity level in anxiety.

**PCA - Subgroup mild anxiety**				
**Variable**	**Component**			
	**1**	**2**	**3**	**4**
Portions of fruit eaten per day				0.891
Portions of fast-food eaten per day		0.88		
Portions of cereals eaten per day			0.853	
Portions of snacks, sweets, and desserts eaten per day		0.845		
Portions of dairy products eaten per day			0.795	
Portions of legumes eaten per day	0.795			
Portions of vegetables eaten per day	0.786			
Portions of supplements eaten per day	0.695			
Portions of beverages eaten per day	0.648			
**PCA - Subgroup moderate anxiety**				
**Variable**	**Component**			
	**1**	**2**	**3**	
Portions of dairy products eaten per day			0.909	
Portions of fruit eaten per day	0.899			
Portions of cereals eaten per day			0.815	
Portions of vegetables eaten per day	0.813			
Portions of beverages eaten per day		0.72		
Portions of fast-food eaten per day		-0.701		
Portions of legumes eaten per day		-0.765		
**PCA - Subgroup severe anxiety**				
**Variable**	**Component**			
	**1**	**2**	**3**	**4**
Portions of supplements eaten per day				0.92
Portions of legumes eaten per day		0.891		
Portions of dairy products eaten per day		0.87		
Portions of fast-food eaten per day			0.851	
Portions of cereals eaten per day	0.835			
Portions of fruit eaten per day	0.74			
Portions of snacks, sweets, and desserts eaten per day	0.667			
Portions of beverages eaten per day			-0.595	

The table shows the factors that make up each component according to the level of severity (mild, moderate, and severe) for anxiety. The value of each component corresponds to the value of the rotated matrix.

Table S11. Component matrix to severity level in depression.

PCA - Subgroup mild depression

Variable	Component			
	1	2	3	4
Portions of cereals eaten per day	0.937			
Portions of snacks, sweets, and desserts eaten per day				0.935
Portions of dairy products eaten per day	0.848			
Portions of beverages eaten per day		0.8272		
Portions of fast-food eaten per day				0.817
Portions of fruit eaten per day		0.739		
Portions of supplements eaten per day			-0.583	
Portions of vegetables eaten per day			-0.695	
Portions of legumes eaten per day			-0.922	

PCA - Subgroup moderate depression

Variable	Component		
	1	2	3
Portions of legumes eaten per day			0.857
Portions of fast-food eaten per day	0.804		
Portions of fruit eaten per day		0.743	
Portions of beverages eaten per day		0.685	
Portions of supplements eaten per day			-0.718

PCA - Subgroup severe depression

Variable	Component			
	1	2	3	4
Portions of fast-food eaten per day				0.975
Portions of supplements eaten per day		0.929		
Portions of legumes eaten per day			0.923	
Portions of fruit eaten per day		0.905		
Portions of dairy products eaten per day			0.905	
Portions of snacks, sweets, and desserts eaten per day	0.808			
Portions of beverages eaten per day	0.794			
Portions of cereals eaten per day	0.725			
Portions of vegetables eaten per day	0.617			

The table shows the factors that make up each component according to the level of severity (mild, moderate, and severe) for depression. The value of each component corresponds to the value of the rotated matrix.

Table S12. Component matrix to severity level in stress.

PCA - Subgroup mild stress				
**Variable**	**Component**			
	**1**	**2**	**3**	**4**
Portions of cereals eaten per day	0.899			
Portions of snacks, sweets, and desserts eaten per day				0.896
Portions of fruit eaten per day		0.775		
Portions of beverages eaten per day		0.763		
Portions of dairy products eaten per day	0.754			
Portions of fast-food eaten per day				0.721
Portions of vegetables eaten per day		0.697		
Portions of legumes eaten per day			-0.779	
Portions of supplements eaten per day			-0.797	
**PCA - Subgroup moderate stress**				
**Variable**	**Component**			
	**1**	**2**	**3**	**4**
Portions of fast-food eaten per day	0.857			
Portions of fruit eaten per day		0.841		
Portions of supplements eaten per day			0.838	
Portions of legumes eaten per day	0.732			
Portions of cereals eaten per day		-0.788		
Portions of dairy products eaten per day		-0.916		
**PCA - Subgroup severe stress**				
**Variable**	**Component**			
	**1**	**2**	**3**	**4**
Portions of dairy products eaten per day		0.887		
Portions of fast-food eaten per day			0.809	
Portions of fruit eaten per day	0.798			
Portions of vegetables eaten per day	0.779			
Portions of cereals eaten per day	0.777			
Portions of legumes eaten per day		0.774		
Portions of snacks, sweets, and desserts eaten per day			0.715	

The table shows the factors that make up each component according to the level of severity (mild, moderate, and severe) for stress. The value of each component corresponds to the value of the rotated matrix.

Table S13. Kaiser–Meyer–Olkin (KMO) measure of sampling adequacy and Bartlett's test of sphericity for food group data across anxiety, and depression levels.

**Variable**		**Category(s)**	**KMO**	**Barlett χ^2**	***P*-value**
**Mental health**	Anxiety	Mild	0.326	65.696	**0.0018**
		Moderate	0.490	73.68	**<0.001**
		Severe	0.580	55.91	**0.018**
	Depresssion	Mild	0.360	72.812	**<0.001**
		Moderate	0.491	49.66	0.064
		Severe	0.383	74.71	**<0.001**

The table presents the results of the KMO measure of sampling adequacy and Bartlett's test of sphericity for food consumption patterns according to severity level (mild, moderate, severe) for anxiety and depression. $P < 0.05$ are highlighted in bold.

Table S14. Kaiser–Meyer–Olkin (KMO) measure of sampling adequacy and Bartlett's test of sphericity for food group data across stress levels.

**Variable**		**Category**	**KMO**	**Barlett χ^2**	***P* value**
**Mental health**	Stress	Mild	0.385	67.957	**0.001**
		Moderate	0.601	66.543	**0.0014**
		Severe	0.611	44.675	0.152

The table presents the results of the KMO measure of sampling adequacy and Bartlett's test of sphericity for food consumption patterns according to severity level (mild, moderate, severe) for stress. $P < 0.05$ are highlighted in bold.

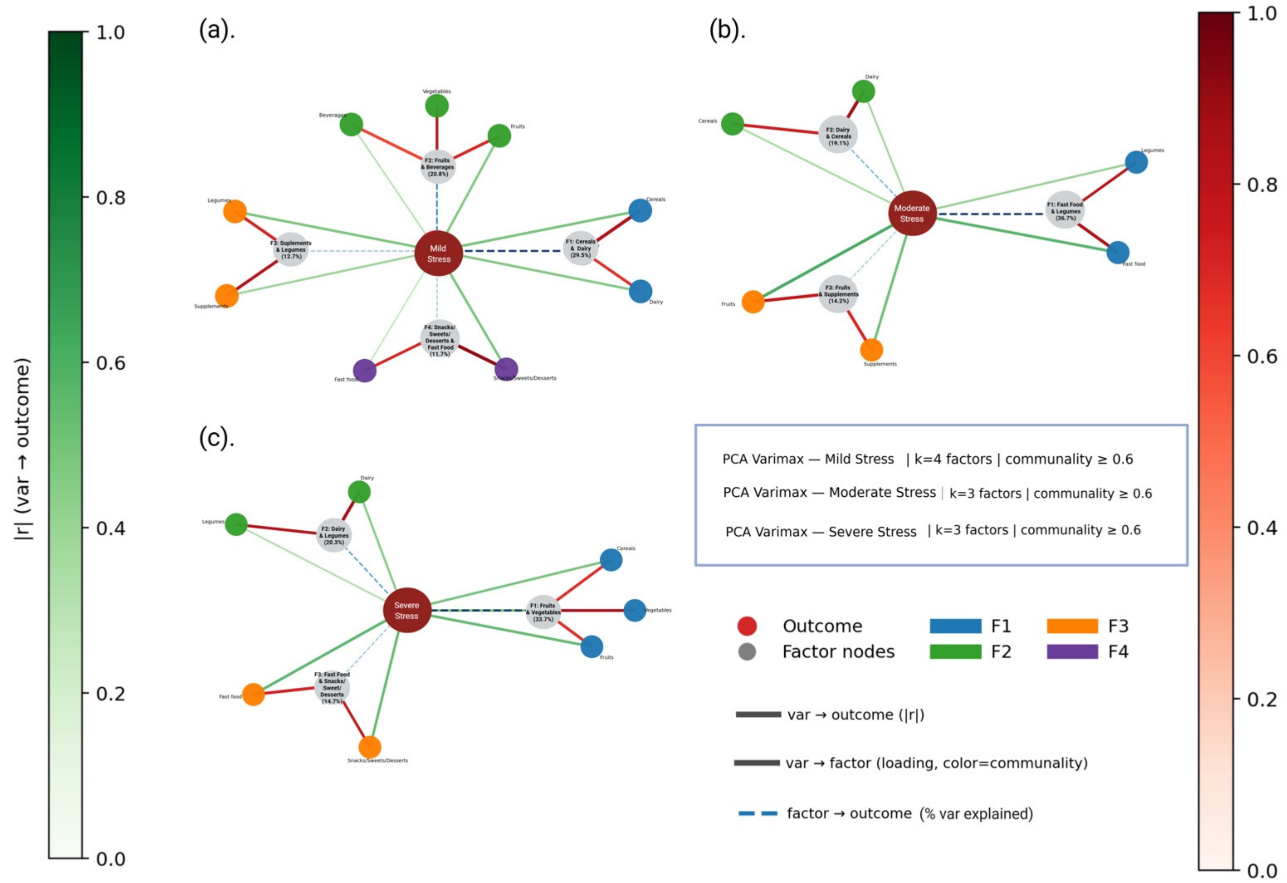

Figure S1. PCA–Varimax network graphs for mild (a), moderate (b), and severe (c) stress. Nodes represent food groups (blue), latent dietary factors (grey), and the stress subgroup outcome (red). Green edges indicate direct associations between food groups and outcome ($|r|$), red edges represent loadings from food groups to factors ($|\lambda|^2$), and dashed blue edges correspond to outcome–factor connections weighted by explained variance.
